# Reduced genomic tumor heterogeneity after neoadjuvant chemotherapy is related to favorable outcome in patients with esophageal adenocarcinoma

**DOI:** 10.18632/oncotarget.9857

**Published:** 2016-06-06

**Authors:** Askar Obulkasim, Bauke Ylstra, Hendrik F. van Essen, Christian Benner, Sally Stenning, Ruth Langley, William Allum, David Cunningham, Imran Inam, Lindsay C. Hewitt, Nicolas P. West, Gerrit A. Meijer, Mark A. van de Wiel, Heike I. Grabsch

**Affiliations:** ^1^ Department of Epidemiology and Biostatistics, VU University Medical Center, Amsterdam, NL; ^2^ Department of Pathology, VU University Medical Center, Amsterdam, NL; ^3^ MRC Clinical Trials Unit at University College London, London, UK; ^4^ Department of Surgery, Royal Marsden NHS Foundation Trust, London, UK; ^5^ Department of Gastrointestinal Oncology, Royal Marsden NHS Foundation Trust, London and Surrey, UK; ^6^ Section of Pathology and Tumour Biology, Leeds Institute of Cancer and Pathology, University of Leeds, Leeds, UK; ^7^ Department of Mathematics, VU University, Amsterdam, NL; ^8^ Department of Pathology and GROW School for Oncology and Developmental Biology, Maastricht University Medical Center, Maastricht, NL

**Keywords:** esophageal adenocarcinoma, array CGH, prognosis, chemotherapy

## Abstract

Neoadjuvant chemo(radio)therapy followed by surgery is the standard of care for patients with locally advanced resectable esophageal adenocarcinoma (EAC). There is increasing evidence that drug resistance might be related to genomic heterogeneity. We investigated whether genomic tumor heterogeneity is different after cytotoxic chemotherapy and is associated with EAC patient survival. We used arrayCGH and a quantitative assessment of the whole genome DNA copy number aberration patterns (‘DNA copy number entropy’) to establish the level of genomic tumor heterogeneity in 80 EAC treated with neoadjuvant chemotherapy followed by surgery (CS group) or surgery alone (S group). The association between DNA copy number entropy, clinicopathological variables and survival was investigated.

DNA copy number entropy was reduced after chemotherapy, even if there was no morphological evidence of response to therapy (p<0.001). Low DNA copy number entropy was associated with improved survival in the CS group (p=0.011) but not in the S group (p=0.396).

Our results suggest that cytotoxic chemotherapy reduces DNA copy number entropy, which might be a more sensitive tumor response marker than changes in the morphological tumor phenotype. The use of DNA copy number entropy in clinical practice will require validation of our results in a prospective study.

## INTRODUCTION

Pre-operative or peri-operative 5-fluorouracil/cisplatin based chemotherapy as well as pre-operative chemoradiotherapy followed by radical resection are the standard of care for patients with locally advanced, resectable esophageal adenocarcinoma (EAC) [1–3]. However, only 20 to 30% of EAC patients currently achieve a durable remission after multimodality treatment [4, 5]. Thus, there is an urgent clinical need to better understand the molecular processes that are involved in disease progression and resistance to therapy.

Cytotoxic chemotherapy exerts one of the strongest selection pressures on cancer cells implying that chemotherapy treatment may favor the persistence of treatment resistant subpopulations [6].

It has been suggested that drug resistance might be related to genomic tumor heterogeneity [7]. High levels of genomic tumor heterogeneity in pre-treatment biopsies have been related to poor response to chemotherapy in EAC [8], as well as been related to poor outcome in head and neck cancers [9] and adenocarcinomas of the lung [10]. However, when measured in paired samples taken before and after chemotherapy, results are variable showing no change in genomic tumor heterogeneity in breast and ovarian cancer [11, 12], decreasing genomic tumor heterogeneity in acute myeloid leukemia [13] and newly acquired genomic changes in acute lymphoblastic leukemia [14].

Several studies have demonstrated the existence of genomic and/or phenotypic tumor heterogeneity in EAC [15–20]. The presence of genomic heterogeneity in Barrett's esophagus, an endoscopically identifiable precursor lesion of EAC, is predictive of progression to adenocarcinoma [17], and its presence in pre-treatment EAC biopsies has been related to poor chemotherapy response [8]. The relationship between genomic tumor heterogeneity and patient outcome and the effect of cytotoxic chemotherapy on genomic tumor heterogeneity have not been investigated in EAC to date.

Whole genome copy number profiling using array comparative genomic hybridization (aCGH) can provide a snapshot of the global DNA copy number aberration pattern. In this hypothesis-generating study, we used aCGH to quantify the degree of genomic tumor heterogeneity within a group of samples by estimating the DNA copy number entropy [21]. This allowed us to explore the differences in the DNA copy number entropy between EAC patients from the OE02 trial treated with neoadjuvant chemotherapy (CS group) and those treated by surgery alone (S group), and to examine the association of DNA copy number entropy with clinicopathological variables, histopathologically measured tumor regression grade according to Mandard and patient survival.

## RESULTS

### Relationship between DNA copy number entropy, treatment group and histopathological tumor regression grade

**Figure 1:**
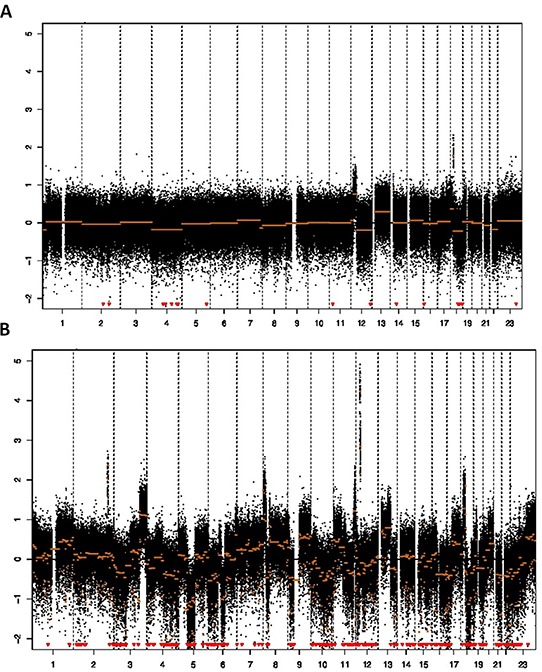
Array CGH profile and DNA copy number entropy **A.** Low DNA copy number entropy case showing a profile with relative few aberrations (sample no. 74, y-axis: log2 ratio of DNA signal from the tumor to matched normal, x-axis: chromosomes in numerical order; DNA copy number entropy: −1.541766, MAD value 0.34), **B.** High DNA copy number entropy case showing many aberrations (gains/amplifications as well as losses) (sample no. 91, y-axis: log2 ratio of DNA signal from the tumor to matched normal, x-axis: chromosomes in numerical order; DNA copy number entropy: 0.5224006, MAD value: 0.33). Note that both samples have similar MAD values (estimates of the noise) indicating that there was no technical difference in the quality of the array CGH experiment.

This study used DNA copy number entropy as a surrogate marker of genomic tumor heterogeneity. A representative DNA copy number profile with low DNA copy number entropy value and one with a high DNA copy number entropy value are shown in Figure 1A and 1B, respectively.

**Figure 2:**
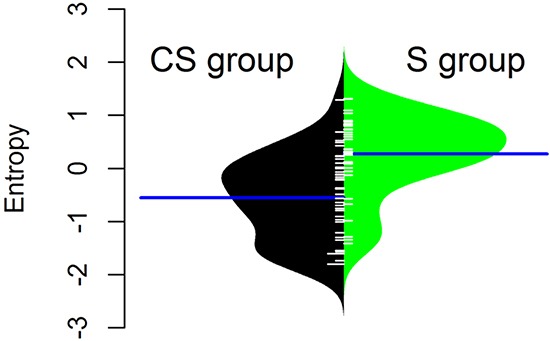
Normalised DNA copy number entropy distribution by treatment group The polygon shape of the bean plot shows the distribution of the DNA copy number entropy values within each group. The DNA copy number entropy distribution is significantly different between CS and S (p< 0.001) with a higher mean DNA copy number entropy in the S group. Patients treated by surgery alone (S group, right side of the panel), patients treated by chemotherapy and surgery (CS group, left side of the panel). Line perpendicular to the long axis of the panel = mean DNA copy number entropy/group; DNA copy number entropy values on the y axis.

**Figure 3:**
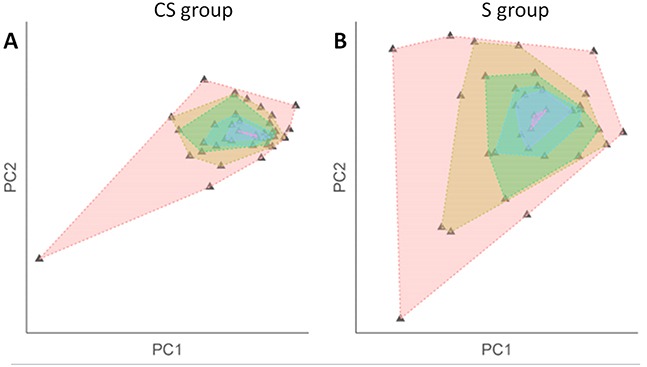
Scatterplot after principal component analysis of the DNA copy number profile by treatment group Plotting the segmented DNA copy number profiles using its principal components shows inter-tumor variation within each treatment group (**A.** CS group; **B.** S group). Each triangle denotes a sample. Each shaded area was created by connecting samples with the same variation level. The smaller the shaded area, the more similar the samples within the group. The scatter plots show that there is less inter-tumor variation in the CS group which is indicative of a higher level of inter-tumor homogeneity reflected in a low DNA copy number entropy value. In the surgery alone group, sample values are more spread out in the plot indicating a higher degree of inter-tumor variation reflected in a higher DNA copy number entropy value.

EAC in the CS group had a significantly lower DNA copy number entropy value compared to EAC in the S group (median DNA copy number entropy (range) CS group: −0.524 (−1.801 to 1.289) versus S group: 0.321 (−1.411 to 1.315), p<0.001, Figure 2), indicating that EACs in the CS group had DNA copy number aberrations at relatively similar genomic locations. The differences and similarities in DNA copy number profiles between the CS and the S group can also be seen in a principal component scatter plot, Figure 3. These plots show that the DNA copy number values (as summarized by their first two principal components) from the S group were much more spread out (Figure 3B) than those from the CS group (Figure 3A), indicating that the variation of the DNA copy number aberrations between EACs in the S group is higher than between EACs in the CS group. In other words, the inter-tumor heterogeneity of the aCGH profile is reduced in EACs after chemotherapy.

The comparison of the DNA copy number entropy values between the CS group and the S group stratified by chromosome showed that DNA copy number entropy values for chromosome 1 and chromosome 5 were significantly different. Median DNA copy number entropy (range) of chromosome 1 was −0.296 (−4.165 to 1.213) in the CS group compared to 0.483 (−3.070 to 1.645) in the S group, p=0.001. Similarly, the median DNA copy number entropy (range) of chromosome 5 was −0.271 (−3.800 to 1.255) in the CS group compared to 0.413 (−3.238 to 1.647) in the S group, p=0.03. However, related to the very high complexity of the aCGH profiles, we were not able to identify specific probes or genes neither on chromosome 1 nor on chromosome 5 which were significantly different between the treatment groups after applying rigorous multiple testing corrections. For all other chromosomes, the DNA copy number entropy values were not statistically significantly different between the CS and the S group (Supplementary Figure S5 and S6).

**Figure 4:**
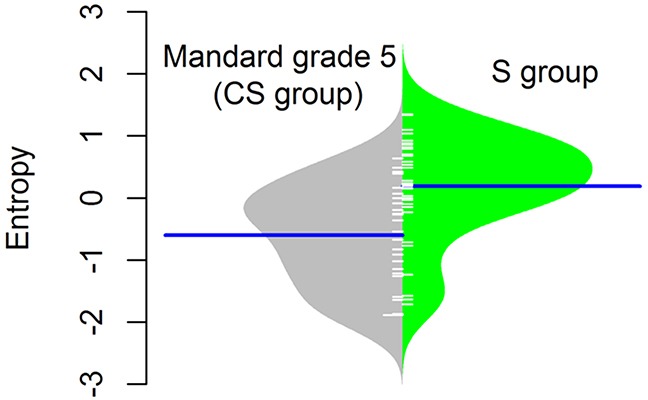
Normalised DNA copy number entropy distribution comparing the surgery alone (S group) samples with no pathological response (Mandard tumor regression grade 5) with samples after chemotherapy (CS group) The polygon shape of the bean plot shows the distribution of the DNA copy number entropy values within each group. The DNA copy number entropy of EAC from patients treated by surgery alone (right side of the panel) is significantly higher than that of EAC treated with neoadjuvant chemotherapy showing no histopathological evidence of tumor regression (left side of the panel), p<0.001. Line perpendicular to the long axis of the panel = mean DNA copy number entropy/group; DNA copy number entropy values on the y axis.

Although the median DNA copy number entropy values increased with increasing Mandard tumor regression grade category e.g. the highest DNA copy number entropy values were seen in EAC with no histological evidence of tumor regression (Mandard grade 5), this relationship was not statistically significant (Mandard grade 3 median DNA copy number entropy: −1.597; Mandard grade 4: −0.696; Mandard grade 5: −0.292, p > 0.1). The lack of statistical significance could be related to the relatively small sample size in the current study. Interestingly, the DNA copy number entropy values of EACs in the S group were significantly higher than the DNA copy number entropy values of EACs in the CS group with no histological evidence of tumor regression (Mandard grade 5, n=26) (median (range) DNA copy number entropy of Mandard grade 5 EACs in the CS group: −0.292 (−1.801 to 0.685), median (range) DNA copy number entropy in the S group: 0.321 (−1.411 to 1.315), p< 0.001, Figure 4).

### Relationship between DNA copy number entropy, clinicopathological variables and patient survival

No association was found between depth of tumor invasion (pT/ypT), lymph node status (pN/ypN), grade of differentiation, lymphatic channel invasion, venous invasion or perineural invasion, and DNA copy number entropy neither when investigating these associations by treatment arm nor in the combined group (Supplementary Table S1).

**Figure 5:**
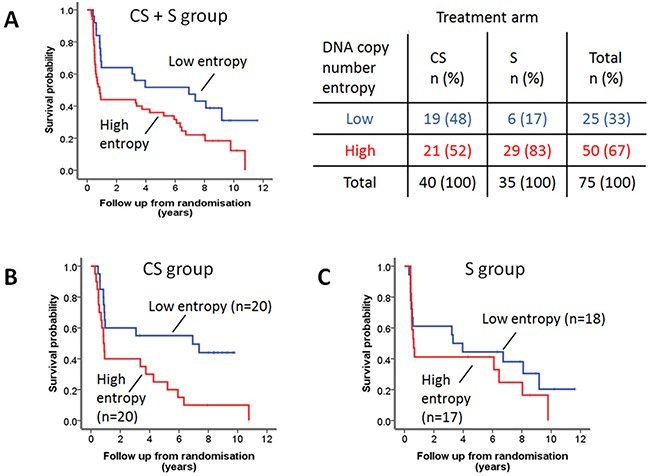
DNA copy number entropy and cancer specific survival **A.** Kaplan-Meier survival analysis of the combined patient cohort. Patients with low DNA copy number entropy (n=27), dichotomized using the median (−0.5670) as a cut-off, have a significantly longer survival than those with high DNA copy number entropy (n=50). Median (95% CI) survival time low DNA copy number entropy 6.94 years (0.56-13.28 years) versus high DNA copy number entropy 0.85 years (0.56-1.14 years), log rank p=0.024. More than 80% of S patients have tumors with high DNA copy number entropy (see crosstable). **B.** Kaplan-Meier survival analysis of the CS group patients. DNA copy number entropy dichotomised at the median. CS patients with low DNA copy number entropy have a significantly longer survival than those with high DNA copy number entropy (median (95% CI) survival time low DNA copy number entropy 6.94 years (0-15.59) vs. high DNA copy number entropy 0.86 years (0.74-0.971), log rank p=0.011). **C.** Kaplan-Meier survival analysis of the S group patients. DNA copy number entropy dichotomised at the median. No relationship was seen between DNA copy number entropy and survival in S patients (log rank p=0.396).

As a consequence of our case selection (selecting short-term and long-term survivors in both groups, see Material and Methods), the survival was not significantly different between the S and CS group. Thus, initial survival analyses were performed combining the two groups. Low DNA copy number entropy was significantly associated with longer cancer specific survival in the whole patient group (HR: 1.382, 95%CI: 1.014-1.884, p=0.041). For Kaplan-Meier survival analysis, three equal sized patient groups were created based on the DNA copy number entropy values. As the survival curves of two of these groups overlapped substantially (Supplementary Figure S4), these two groups were subsequently merged. Kaplan-Meier survival analysis showed that the group of patients with low DNA copy number entropy (≤ −0.5670), e.g. with DNA copy number aberrations at similar genomic locations, survived significantly longer than patients with high DNA copy number entropy, e.g. DNA copy number aberration at multiple different genomic locations, p=0.024, Figure 5A. Cross tabulation between DNA copy number entropy group and treatment group indicated that 29 (83%) S group patients fell into the high DNA copy number entropy group (entropy > −0.5670), whereas patients from the CS group were equally distributed between the high and low DNA copy number entropy group (Figure 5). This observation prompted us to further explore the relationship between DNA copy number entropy and survival by treatment arm.

Cox regression analysis using continuous DNA copy number entropy values showed that DNA copy number entropy was significantly associated with cancer specific survival in the CS group (HR: 1.775, 95% CI: 1.047-3.009, p=0.033) but not in the S group (HR: 1.144, 95%CI: 0.699-1.871, p=0.593). For visualization of the Cox regression analysis results, Kaplan-Meier survival plots were created stratifying patients by using the median DNA copy number entropy per treatment group as cut-off. CS group patients with low DNA copy number entropy had a significantly better survival (median (range) survival time: 6.9 years (0 to 15.6 years)) compared to CS group patients with high DNA copy number entropy (0.9 years (0.7 to 0.97 years), p=0.011, Figure 5B). No relationship between DNA copy number entropy and survival was found in the S group patients (p=0.396, Figure 5C). Cox regression analysis showed that there is no evidence of a treatment interaction effect (treatment interaction p=0.654). Hence, we have no evidence that the effect of the DNA copy number entropy depends on the treatment.

### Comparison of the DNA copy number profile and DNA copy number entropy with previous studies

**Figure 6:**
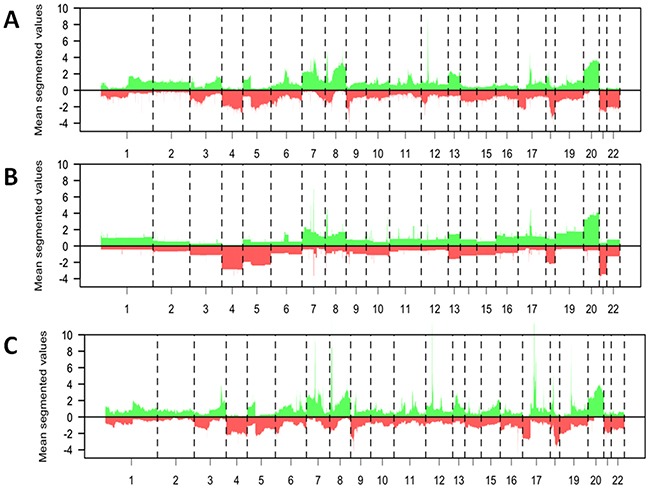
Comparison of the data from the surgery alone group of the current study with previously published datasets from patients with esophageal adenocarcinoma.**A–C** Mean DNA copy number aberrations plotted by chromosome position (x-axis) using segmented data points present in all datasets. Gains = above the 0 line, loss = below the 0 line. The profile of the DNA copy number data from the S group patients from the current study (A, n=35) is visually similar to that previously published by Goh et al (B, n=53) and to that previously published by Dulak et al. (C, n=74, 16321 probes).

In order to assess whether the aCGH profiles and DNA copy number entropy of EAC of the current study are comparable to previously published EAC DNA copy number data, the frequency of DNA copy number aberrations and DNA copy number entropy of the S group EACs were compared to two recently published data sets [22, 23]. Visual inspection of the DNA copy number aberration frequency plots from all three studies was performed and profiles were deemed to be similar on visual inspection (Figure 6A to 6C).

### Relationship between DNA copy number entropy and percentage of tumor cells/area

EAC from the CS group had a median of 70% tumor cells/area ranging from 30 to 90% (Supplementary Figure S2). EAC from the S group had a median of 50% tumor cells/area ranging from 30 to 70%. There was no association between the DNA copy number entropy and the estimated tumor percentage/area neither in the CS group nor the S group (p=0.412 and p=0.531, respectively). There was also no significant difference in the percentage of tumor cells/area between the long-term and short-term survivors in any of the groups (CS: p=0.103; S: p=0.439). We observed neither considerable differences in scatterness between the high and the low tumor cells/area groups nor differences in scatterness when each treatment group was analyzed separately (Supplementary Figure S3).

## DISCUSSION

There have been a number of DNA copy number studies in esophageal adenocarcinoma (EAC) in the past, reporting different candidate genes or group of genes related to tumor progression or patient survival [18–20, 23–26]. A common finding of all previous EAC studies as well as the current study is that the overall level of DNA copy number alterations in EAC was very high leading to very complex DNA copy number profiles. However, only few aberrations were shared across more than 10% of samples supporting the existence of a high degree of genomic tumor heterogeneity in EAC.

The frequency of DNA copy number aberrations (irrespective of the genes involved) has been related to survival in EAC in some but not all studies [23–25]. The current study is the first study to quantify the DNA copy number entropy by whole genome aCGH as a surrogate measure of genomic inter-tumor heterogeneity using DNA extracted from resection material from patients who were either treated by surgery alone (S group) or by 5FU/cisplatin chemotherapy followed by surgery (CS group) as part of the OE02 trial [1].

We found no relationship between DNA copy number entropy and any of the investigated clinicopathological data neither in the S group nor in the CS group. Importantly, there was also no statistically significant association between DNA copy number entropy and Mandard tumor regression grade of the primary tumor (only investigated in the CS group) or percentage of tumor/area (investigated in both groups). These results provide some confidence that the DNA copy number entropy is not simply a surrogate of some other pathological variable or known prognostic factor. Our findings are consistent with results reported in head and neck cancer where the variability of the mutant-allele fraction was used as marker of genomic tumor heterogeneity [9].

Our study showed that EACs after chemotherapy have DNA copy number profiles in which the aberrations were found more frequently at relatively similar locations, making them more ‘homogenous’ when measured by DNA copy number entropy compared to chemo-naïve EACs (Figure 3). In contrast, Almendro *et al* [11] reported a change in the morphological but not the genomic phenotype of the tumor cells in breast cancer after chemotherapy. Our study showed no difference in the morphological tumor phenotype comparing EAC from the S group with those from the CS group with Mandard grade 5 (no pathological response). However, we found a significant difference in the genomic phenotype between Mandard grade 5 EACs from the CS group and EACs from the S group. This discrepancy between studies could be related to different tumor types (EAC vs. breast cancer) as well as substantial differences in investigated markers (whole genome vs. few selected markers) used to characterize the tumor phenotype and genotype. Further studies in a larger sample size need to verify whether DNA copy number entropy might be a clinically useful biomarker of response to chemotherapy.

Our analyses comparing the DNA copy number entropy per chromosome between treatment groups showed that the difference in the DNA copy number entropy was strongly associated with the DNA copy number entropy in chromosome 1 and 5. The underlying mechanisms for this finding are currently unclear and warrant future studies. So far, there is no evidence from previous aCGH studies suggesting clinically relevant candidate genes on these particular chromosomes in EAC and we were unable to identify any individual significantly different probe or group of genes on these chromosomes in the current study.

Low DNA copy number entropy was only related to better outcome in EAC patients who had received chemotherapy prior to surgery. Mroz *et al* [9] reported the relation between tumor heterogeneity and patient outcome in head and neck cancer patients treated by surgery alone as well as in those treated with chemotherapy. Murugaesu et al [8] reported that intra-tumoral genomic heterogeneity of the pre-treatment biopsy is related to poor response to platinum based chemotherapy. However, both studies determined genomic tumor heterogeneity prior to chemotherapy, whereas we measured DNA copy number in material after chemotherapy, hence results are not directly comparable.

We compared the aCGH profiles and the DNA copy number entropy of the surgery alone group with published data sets [22, 23]. Visual inspection showed that major aberrations like copy number losses in chromosome (Chr) 3, gains in Chr7, Chr8 and Chr20 were present in all studies. Hence, we think that findings in this study were unlikely to be dataset specific. Due to difference in platforms and hence differences in data resolution, statistical assessment and quantification of the differences were not possible. Since, post-chemotherapy aCGH data from EAC have not been published to date, no comparison to previous data could be made for this group.

In conclusion, this is the first study to quantify genomic tumor heterogeneity in esophageal adenocarcinoma, and to suggest that it might contribute to survival differences after chemotherapy.

Our result requires validation in a larger independent cohort ideally comparing pre- and post-chemotherapy samples from the same tumor to directly measure the change in DNA copy number entropy induced by chemotherapy. The presence of genomic tumor heterogeneity, together with low frequencies of common aberrations across multiple cancers, makes the identification of predictive/prognostic biomarkers in EAC challenging, unless DNA copy number entropy itself can be used for this purpose. For the first time, we can show that cytotoxic chemotherapy appears to effect the tumor genotype (DNA copy number) in cases where the human eye cannot see any changes in the histological phenotype, suggesting that we might need to re-evaluate and potentially adapt the morphology based assessment criteria of primary tumor regression grading in particular in cases with a high tumor cell content per area.

A better understanding of the mechanisms leading to genomic tumor heterogeneity is urgently needed before novel therapeutic strategies can be developed and outcome of EAC patients can finally be improved.

## MATERIALS AND METHODS

### Esophageal adenocarcinoma patient cohort

The UK MRC OE02 trial recruited 802 patients with locally advanced esophageal cancer who were randomized to either two cycles of cisplatin/5-fluorouracil (5-FU) followed by surgery (CS group) or treatment by surgery alone (S group) between 1992 and 1998 [1]. Formalin-fixed and paraffin-embedded (FFPE) tissue blocks from the resection specimens were retrospectively collected from 510 OE02 trial patients. For this pilot study, 40 patients from the CS group and 40 patients from the S group were selected based on the availability of appropriate material for DNA extraction and the following criteria: (1) presence of adenocarcinoma in the pre-treatment biopsy, (2) CS patients were known to have received at least one cycle of pre-operative chemotherapy, (3) patients were reported to have died from esophageal cancer. In order to increase the chance to detect potential prognostic markers within treatment groups, patients were further selected based on their survival time to ensure that in each treatment group 50% patients that had died within 12 months after randomization (short-term survivors) and 50% had survived at least 36 months after randomization (long-term survivors). Consequently, this selection resulted in a data set with large differences in survival within treatment groups and no survival difference between treatment groups (p=0.62). Median (range) follow up of short-term survivors was 0.72 years (0.28 to 0.97 years) and 0.46 years (0.30 to 0.67 years) in the CS and S group, respectively. Median (range) follow up of long-term survivors was 7.65 years (3.07 to 10.76 years) and 7.30 years (3.23 to 11.62 years) in the CS and S group, respectively. The median age (range) was 60.1 years (39.8 to 83.15 years) and 65.13 years (40.87 to 75.77 years) in the CS and S group, respectively.

The hematoxylin and eosin stained glass slides from all retrieved resection specimens and the original histopathology reports were reviewed to establish the histopathology data set used for analyses. Regression of the primary tumor was graded according to Mandard *et al* [27] centrally. Cases were staged according to the 6th edition of the TNM classification [28]. Details of the clinicopathological data of the patient cohort can be found in Table 1.

**Table 1:** Clinicopathological data by treatment arm (CS: chemotherapy + surgery; S: surgery alone)

	CS n (%)	S n (%)
	40 (53)	35 (47)
Gender		
Female	8 (20)	4 (11)
Male	32 (80)	31 (89)
Number of deaths due to cancer	30 (75)	27 (77)
Depth of tumor invasion (pT/ypT)		
T1	7 (19)	4 (11)
T2	2 (5)	4 (11)
T3	31 (76)	27 (78)
Lymph node status (pN/ypN)		
N0	13 (33)	7 (20)
N1	27 (67)	28 (80)
Lymphatic channel invasion		
Absent	27 (67)	15 (43)
Present	13 (33)	20 (57)
Blood vessel invasion		
Absent	36 (90)	28 (80)
Present	4 (10)	7 (20)
Peri- or intraneural invasion		
Absent	25 (62)	13 (37)
Present	15 (38)	22 (63)
Grade of differentiation (worst)^#^		
Well	3 (8)	0
Moderate	13 (33)	6 (17)
Poor	23 (58)	29 (83)
Mandard tumor regression grade^*^		
3	3 (7)	−
4	11 (28)	
5	26 (65)	

# one missing value in the CS group.

* tumor regression grading not applicable for surgery alone cases.

### DNA extraction and DNA copy number analysis

The tumor cell content per macrodissected tumor tissue area was estimated. As it had been shown previously that DNA copy number analyses from tumors with less than 30% tumor cells/area can be severely compromised [29], five cases (all from the S group) with less than 30% tumor cells/area had to be excluded from further analyses. DNA was extracted from a single FFPE tissue block containing the highest tumor cell density per area as well as from normal tissue (lymph node or normal esophageal wall) from the same patient as described previously [30].

High-resolution array comparative genomic hybridization (aCGH) was performed using custom design 180K Agilent microarrays (4×180k array, Agilent Technologies, Palo Alto, CA, USA, GEO platform GPL8687) containing 180,880 *in situ* synthesized oligonucleotides representing 169,793 unique chromosomal locations evenly distributed over the genome (spacing ~17 kb) and 4548 additional unique oligonucleotides, located at 238 of the Cancer Census genes (http://www.sanger.ac.uk/genetics/CGP/Census/).

Oligonucleotide positions were defined according to the NCBI36/hg18 assembly (March 2006). Labeling and hybridization were performed as described previously by Buffart *et al* [31]. Agilent's feature extraction software (v10.5.1.1) with default settings was used to quantify the fluorescence intensities of all 180,880 features. aCGH data have been deposited in NCBI's Gene Expression Omnibus and are accessible through GEO accession number GSE56106.

### aCGH data pre-processing

aCGH data were generated from 75 cases. The raw data underwent a series of pre-processing steps, which are detailed in Section S1 of the Supplementary file. The pre-processing was performed within the R statistical environment. After quantification, control spots (n=6,539) were removed from the data yielding 174,341 features. Both channels were background corrected by subtracting background median intensities from foreground median intensities to obtain an unbiased hybridization signal. Normalization, cellularity correction and segmentation were performed with the R-package CGHcall [32]. The median absolute deviation (MAD) was calculated for all probes from all chromosomes as described by [33] as a measure of the technical quality of the aCGH experiment. All statistical analyses were performed using segmented data.

### Statistical analyses

DNA copy number profiles of the patients included in the current study were compared with two previously published DNA copy number data sets (Gene Expression Omnibus (GEO) database accession number: GSE36460 and GSE20154) [22, 23]. All raw datasets were pre-processed and segmented following the same procedures as used in this study. The genomic locations of CGH probes across studies were matched using the R-package sigaR [34] and a mean segmented data profile (average across samples) was generated for each data set (Supplementary Figure S1).

Next, we quantified the degree of genomic tumor heterogeneity for each case by assessing the overall DNA copy number aberration pattern and estimating the DNA copy number entropy using the segmented data and the R-package sigaR [34] (see supplementary file for mathematical description of the entropy calculation). The mean DNA copy number entropy was compared between treatment groups using permutation for p-value calculation, where the test statistic equals the entropy difference between the groups. To visualize the intersample heterogeneity, as measured by the DNA copy number entropy, the segmented data matrix was projected on its first two principal components and scatter plots were generated for each treatment group.

The DNA copy number entropy of a sample without DNA copy number aberrations, such as normal tissue, is very low to minimal. Tumor samples can have near zero, few or many DNA copy number aberrations and, thus, the DNA copy number entropy value can range from minimal/low to high [21]. In order to investigate the potential contribution of an individual chromosome to the observed difference in DNA copy number entropy between treatment groups, the entropy-based data analysis approach was also applied to each chromosome separately.

To establish whether there was any relationship between differences in the DNA copy number entropy and tumor cellularity of the sample, we constructed a linear model with tumor cellularity as confounder. Furthermore, the segmented data profiles of <50% (low cellularity) and >= 50% (high cellularity) samples in each treatment arm were projected on its first two principal components separately, for visual inspection of the difference in scatterness.

The association between DNA copy number entropy and cancer specific survival was investigated using a Cox proportional hazards model. The potential prognostic value of the treatment, in addition to DNA copy number entropy, was investigated in a Cox proportional hazards model.

The associations between DNA copy number entropy and tumor regression grade (CS group only), depth of invasion (pT/ypT), lymph node status (pN/ypN) and grade of differentiation were investigated using the test statistic implemented in the R-package sigaR. P-values <0.05 were considered statistically significant for single tests, and Benjamini-Hochberg corrected false discovery rates (FDR) <0.1 were considered statistically significant for multiple tests. Statistical analyses were performed using R, version 3.0.

The study was approved by the London – South East ethics committee (REC reference: 07/H1102/111).

## SUPPLEMENTARY FIGURES AND TABLE



## References

[1] Allum WH, Stenning SP, Bancewicz J, Clark PI, Langley RE (2009). Long-term results of a randomized trial of surgery with or without preoperative chemotherapy in esophageal cancer. J Clin Oncol.

[2] van Hagen P, Hulshof MC, van Lanschot JJ, Steyerberg EW, van Berge Henegouwen MI, Wijnhoven BP, Richel DJ, Nieuwenhuijzen GA, Hospers GA, Bonenkamp JJ, Cuesta MA, Blaisse RJ, Busch OR, ten Kate FJ, Creemers GJ, Punt CJ (2012). Preoperative chemoradiotherapy for esophageal or junctional cancer. N Engl J Med.

[3] Cunningham D, Allum WH, Stenning SP, Thompson JN, Van de Velde CJ, Nicolson M, Scarffe JH, Lofts FJ, Falk SJ, Iveson TJ, Smith DB, Langley RE, Verma M, Weeden S, Chua YJ, Participants MT (2006). Perioperative chemotherapy versus surgery alone for resectable gastroesophageal cancer. N Engl J Med.

[4] Zacherl J, Sendler A, Stein HJ, Ott K, Feith M, Jakesz R, Siewert JR, Fink U (2003). Current status of neoadjuvant therapy for adenocarcinoma of the distal esophagus. World J Surg.

[5] Burak WE (2003). Is neoadjuvant therapy the answer to adenocarcinoma of the esophagus?. Amercian Journal of Surgery.

[6] Merlo LM, Pepper JW, Reid BJ, Maley CC (2006). Cancer as an evolutionary and ecological process. Nat Rev Cancer.

[7] Saunders NA, Simpson F, Thompson EW, Hill MM, Endo-Munoz L, Leggatt G, Minchin RF, Guminski A (2012). Role of intratumoural heterogeneity in cancer drug resistance: molecular and clinical perspectives. EMBO Molecular Medicine.

[8] Murugaesu N, Wilson GA, Birkbak NJ, Watkins TB, McGranahan N, Kumar S, Abbassi-Ghadi N, Salm M, Mitter R, Horswell S, Rowan A, Phillimore B, Biggs J, Begum S, Matthews N, Hochhauser D (2015). Tracking the genomic evolution of esophageal adenocarcinoma through neoadjuvant chemotherapy. Cancer discovery.

[9] Mroz EA, Tward AD, Pickering CR, Myers JN, Ferris RL, Rocco JW (2013). High intratumor genetic heterogeneity is related to worse outcome in patients with head and neck squamous cell carcinoma. Cancer.

[10] Chen ZY, Zhong WZ, Zhang XC, Su J, Yang XN, Chen ZH, Yang JJ, Zhou Q, Yan HH, An SJ, Chen HJ, Jiang BY, Mok TS, Wu YL (2012). EGFR mutation heterogeneity and the mixed response to EGFR tyrosine kinase inhibitors of lung adenocarcinomas. Oncologist.

[11] Almendro V, Cheng YK, Randles A, Itzkovitz S, Marusyk A, Ametller E, Gonzalez-Farre X, Munoz M, Russnes HG, Helland A, Rye IH, Borresen-Dale AL, Maruyama R, van Oudenaarden A, Dowsett M, Jones RL (2014). Inference of tumor evolution during chemotherapy by computational modeling and in situ analysis of genetic and phenotypic cellular diversity. Cell reports.

[12] Cooke SL, Ng CK, Melnyk N, Garcia MJ, Hardcastle T, Temple J, Langdon S, Huntsman D, Brenton JD (2010). Genomic analysis of genetic heterogeneity and evolution in high-grade serous ovarian carcinoma. Oncogene.

[13] Ding L, Ley TJ, Larson DE, Miller CA, Koboldt DC, Welch JS, Ritchey JK, Young MA, Lamprecht T, McLellan MD, McMichael JF, Wallis JW, Lu C, Shen D, Harris CC, Dooling DJ (2012). Clonal evolution in relapsed acute myeloid leukaemia revealed by whole-genome sequencing. Nature.

[14] Mullighan CG, Phillips LA, Su X, Ma J, Miller CB, Shurtleff SA, Downing JR (2008). Genomic analysis of the clonal origins of relapsed acute lymphoblastic leukemia. Science.

[15] Maley CC, Galipeau PC, Finley JC, Wongsurawat VJ, Li X, Sanchez CA, Paulson TG, Blount PL, Risques RA, Rabinovitch PS, Reid BJ (2006). Genetic clonal diversity predicts progression to esophageal adenocarcinoma. Nat Genet.

[16] Klein CA, Stoecklein NH (2009). Lessons from an aggressive cancer: evolutionary dynamics in esophageal carcinoma. Cancer Res.

[17] Merlo LM, Shah NA, Li X, Blount PL, Vaughan TL, Reid BJ, Maley CC (2010). A comprehensive survey of clonal diversity measures in Barrett's esophagus as biomarkers of progression to esophageal adenocarcinoma. Cancer prevention research.

[18] Dulak AM, Stojanov P, Peng S, Lawrence MS, Fox C, Stewart C, Bandla S, Imamura Y, Schumacher SE, Shefler E, McKenna A, Carter SL, Cibulskis K, Sivachenko A, Saksena G, Voet D (2013). Exome and whole-genome sequencing of esophageal adenocarcinoma identifies recurrent driver events and mutational complexity. Nat Genet.

[19] Ross-Innes CS, Becq J, Warren A, Cheetham RK, Northen H, O'Donovan M, Malhotra S, di Pietro M, Ivakhno S, He M, Weaver JM, Lynch AG, Kingsbury Z, Ross M, Humphray S, Bentley D (2015). Whole-genome sequencing provides new insights into the clonal architecture of Barrett's esophagus and esophageal adenocarcinoma. Nat Genet.

[20] Stachler MD, Taylor-Weiner A, Peng S, McKenna A, Agoston AT, Odze RD, Davison JM, Nason KS, Loda M, Leshchiner I, Stewart C, Stojanov P, Seepo S, Lawrence MS, Ferrer-Torres D, Lin J (2015). Paired exome analysis of Barrett's esophagus and adenocarcinoma. Nat Genet.

[21] van Wieringen WN, van der Vaart AW (2011). Statistical analysis of the cancer cell's molecular entropy using high-throughput data. Bioinformatics.

[22] Dulak AM, Schumacher SE, van Lieshout J, Imamura Y, Fox C, Shim B, Ramos AH, Saksena G, Baca SC, Baselga J, Tabernero J, Barretina J, Enzinger PC, Corso G, Roviello F, Lin L (2012). Gastrointestinal adenocarcinomas of the esophagus, stomach, and colon exhibit distinct patterns of genome instability and oncogenesis. Cancer Res.

[23] Goh XY, Rees JR, Paterson AL, Chin SF, Marioni JC, Save V, O'Donovan M, Eijk PP, Alderson D, Ylstra B, Caldas C, Fitzgerald RC (2011). Integrative analysis of array-comparative genomic hybridisation and matched gene expression profiling data reveals novel genes with prognostic significance in oesophageal adenocarcinoma. Gut.

[24] Frankel A, Armour N, Nancarrow D, Krause L, Hayward N, Lampe G, Smithers BM, Barbour A (2014). Genome-wide analysis of esophageal adenocarcinoma yields specific copy number aberrations that correlate with prognosis. Genes Chromosomes Cancer.

[25] Pasello G, Agata S, Bonaldi L, Corradin A, Montagna M, Zamarchi R, Parenti A, Cagol M, Zaninotto G, Ruol A, Ancona E, Amadori A, Saggioro D (2009). DNA copy number alterations correlate with survival of esophageal adenocarcinoma patients. Mod Pathol.

[26] Paulson TG, Maley CC, Li X, Li H, Sanchez CA, Chao DL, Odze RD, Vaughan TL, Blount PL, Reid BJ (2009). Chromosomal instability and copy number alterations in Barrett's esophagus and esophageal adenocarcinoma. Clin Cancer Res.

[27] Mandard AM, Dalibard F, Mandard JC, Marnay J, Henry-Amar M, Petiot JF, Roussel A, Jacob JH, Segol P, Samama G (1994). Pathologic assessment of tumor regression after preoperative chemoradiotherapy of esophageal carcinoma. Clinicopathologic correlations. Cancer.

[28] International Union Against Cancer (2002). TNM Classification of Malignant Tumours.

[29] Krijgsman O, Israeli D, van Essen HF, Eijk PP, Berens ML, Mellink CH, Nieuwint AW, Weiss MM, Steenbergen RD, Meijer GA, Ylstra B (2013). Detection limits of DNA copy number alterations in heterogeneous cell populations. Cell Oncol (Dordr).

[30] van Essen HF, Ylstra B (2012). High-resolution copy number profiling by array CGH using DNA isolated from formalin-fixed, paraffin-embedded tissues. Methods Molecular Biology.

[31] Buffart TE, Israeli D, Tijssen M, Vosse SJ, Mrsic A, Meijer GA, Ylstra B (2008). Across array comparative genomic hybridization: a strategy to reduce reference channel hybridizations. Genes Chromosomes Cancer.

[32] Wiel MAvd, Kim KI, Vosse SJ, vanWieringen WN, Wilting SM, Ylstra B (2005). CGHcall: an algorithm for calling aberrations for multiple array CGH tumor profiles. Bioinformatics.

[33] Krijgsman O, Israeli D, Haan JC, van Essen HF, Smeets SJ, Eijk PP, Steenbergen RD, Kok K, Tejpar S, Meijer GA, Ylstra B (2012). CGH arrays compared for DNA isolated from formalin-fixed, paraffin-embedded material. Genes Chromosomes Cancer.

[34] van Wieringen WN (2012). Statistics for integrative genomics analyses in R Bioconductor R Package.

