# Supplementary material for: Reduced genomic tumor heterogeneity after neoadjuvant chemotherapy is related to favorable outcome in patients with esophageal adenocarcinoma
Source: Oncotarget. 2016 Jun 6;7(28):44084–95. doi: 10.18632/oncotarget.9857 (PMC5190081; doi:10.18632/oncotarget.9857)
Supplement: Supplementary file 1 [file oncotarget-07-44084-s001.pdf]

Reduced genomic tumor heterogeneity after neoadjuvant chemotherapy is related to favorable outcome in patients with esophageal adenocarcinoma

SUPPLEMENTARY FIGURES AND TABLE

Array CGH data preprocessing

After computing log2 ratios, missing values were imputed using a k-nearest neighbor algorithm implemented in the R-package ‘impute’ available from Bioconductor. Missing values were imputed if values of a particular feature were available from more than 30% of all experiments. By applying this imputation procedure, the total number of features was reduced to 173,367 features. Afterwards, CGH profiles were wave bias corrected by regressing them on a calibration set containing 16 normal profiles to improve detection of aberrations [1]. In a last preprocessing step, microarray data was global median normalized and tumor % corrected using an approach described by van de Wiel et al [2]. Subsequently, copy number profiles were inspected visually. The median cellularity of remaining 75 samples is 60%. The final data matrix that has been used for downstream analysis was of size 173367 × 75. Normalization, tumour cellularity correction and segmentation were performed with the R-package *CGHcall* was used for preprocessing and segmentation.

Mathematical description of the entropy calculation steps

In our setting, entropy measures how far a particular sample is away from the rest in *p*-dimensional genomics space. Specifically, entropy of sample S_i is calculated in the following way: first, the kth-nearest neighbor of S_i is determined, which will be used to estimate the probability density function:

$$f(S_i) = \frac{k}{n-1} V^p \frac{1}{\left(d_k(S_i)\right)^p}$$

where n is the total number of samples, p is the number of features, V^p is the volume of a unit ball in p dimensional space, $d_k(S_i)$ is the Euclidean distance between S_i and its kth nearest neighbor. Finally, the entropy of S_i is equal to

$$H(S_i) = -\log\left(f(S_i)\right)$$

REFERENCES

1. van de Wiel MA, Brosens R, Eilers P, *et al.* Smoothing waves in array CGH tumor profiles. *Bioinformatics* 2009;25:1099-104.
2. van de Wiel MA, Kim K, Vosse SJ, *et al.* CGHcall: calling aberrations for array CGH tumor profiles. *Bioinformatics* 2007;23:892-94.
3. Teschendorff AE, Widschwendter M. Differential variability improves the identification of cancer risk markers in DNA methylation studies profiling precursor cancer lesions. *Bioinformatics* 2012;28:1487-94.

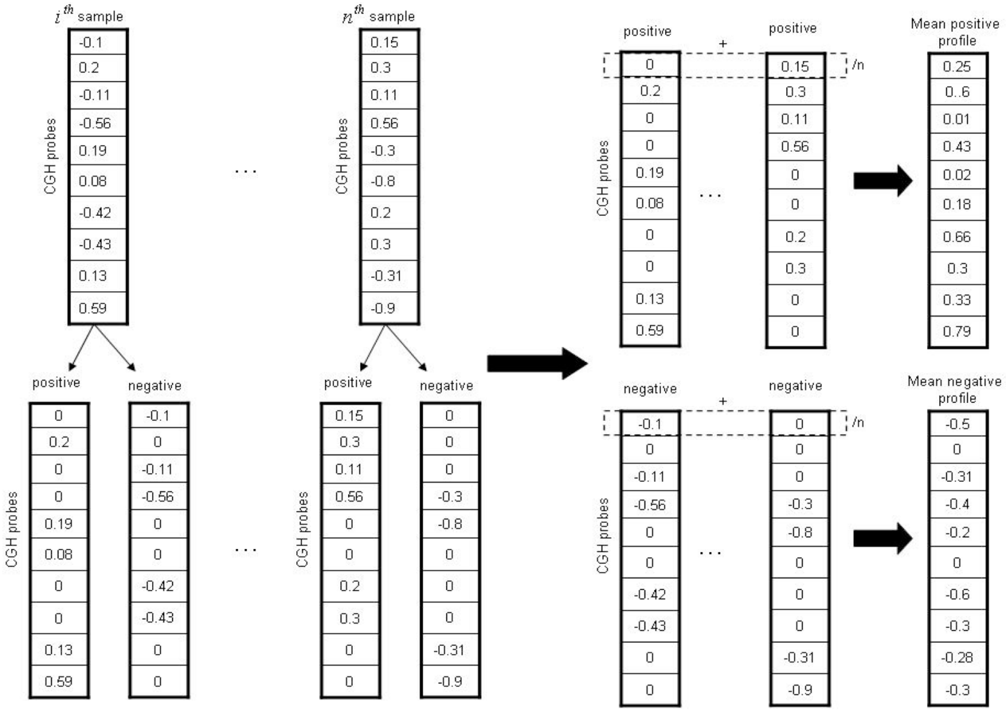

Supplementary Figure S1: Schemata of the averaged CGH profile generation from a given segmented data. For each sample its segmented data values were first divided into positive and negative parts, roughly equal to gain and loss. The positive values across samples were averaged to calculate mean positive segmented values. The mean negatives segmented values were generated in similar ways. Finally, they were plotted together in barplots to illustrate the averaged DNA copy number aberration patterns in a given segmented data.

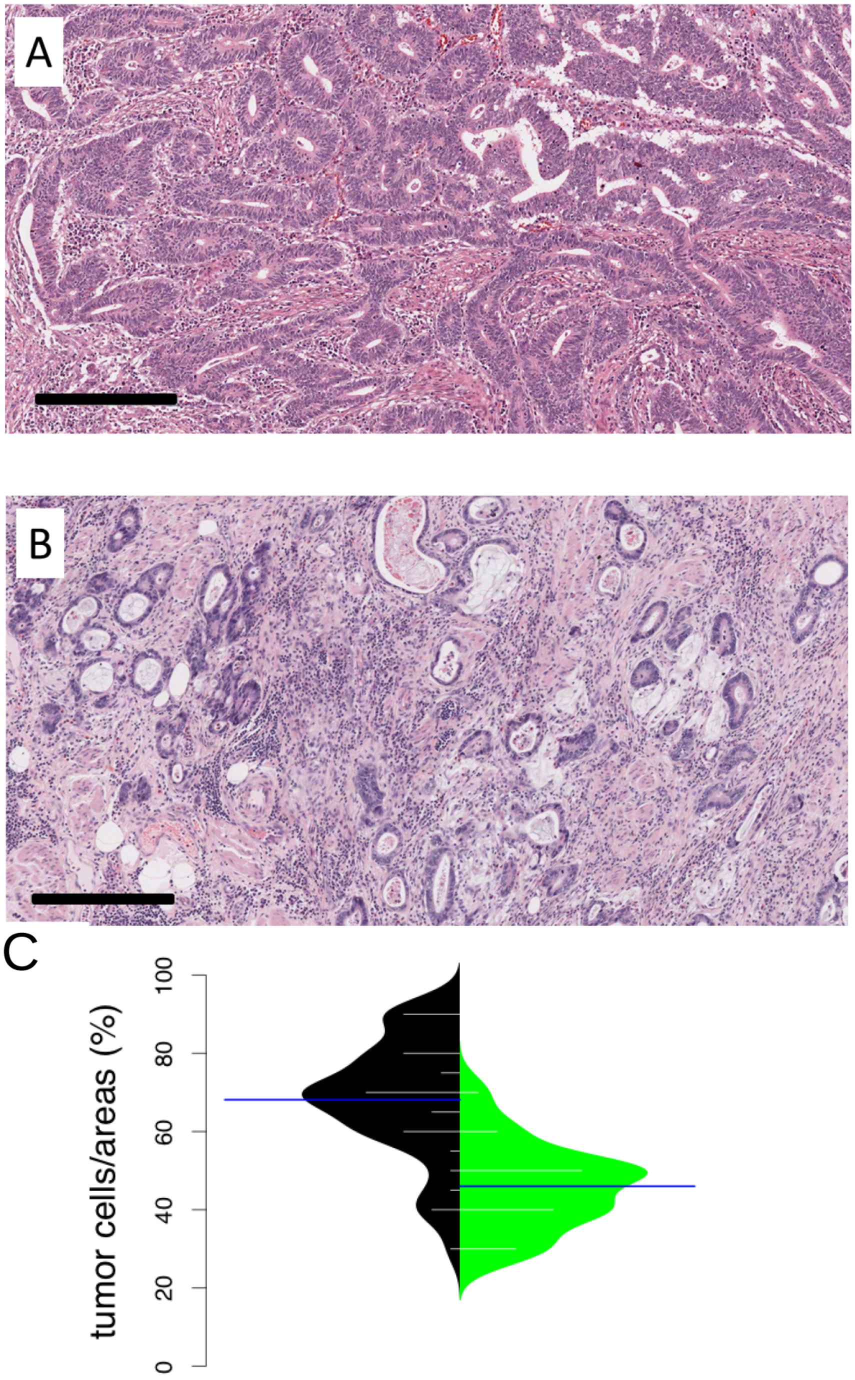

Supplementary Figure S2: Comparing tumor cells/area between treatment groups. A. Example of HE stained section with high number of tumor cells/area (85%). Bar = 300μm **B.** Example of HE stained section with low number of tumor cells/area (20%) due to the infiltration of the tumor with many inflammatory cells. Bar = 300 μm Note that all HE stained slide from the cases used in this study have been scanned and are viewable via the internet upon request to H.Grabsch@maastrichtuniversity.nl. **C.** Bean plot comparing the tumor cells/areas distribution between the treatment groups. The shape and the mean (blue line) of tumor cells/areas are relatively different between the treatment groups. S (right) appears to be composed of samples with low tumor cells/area whereas the opposite holds true for CS (left).

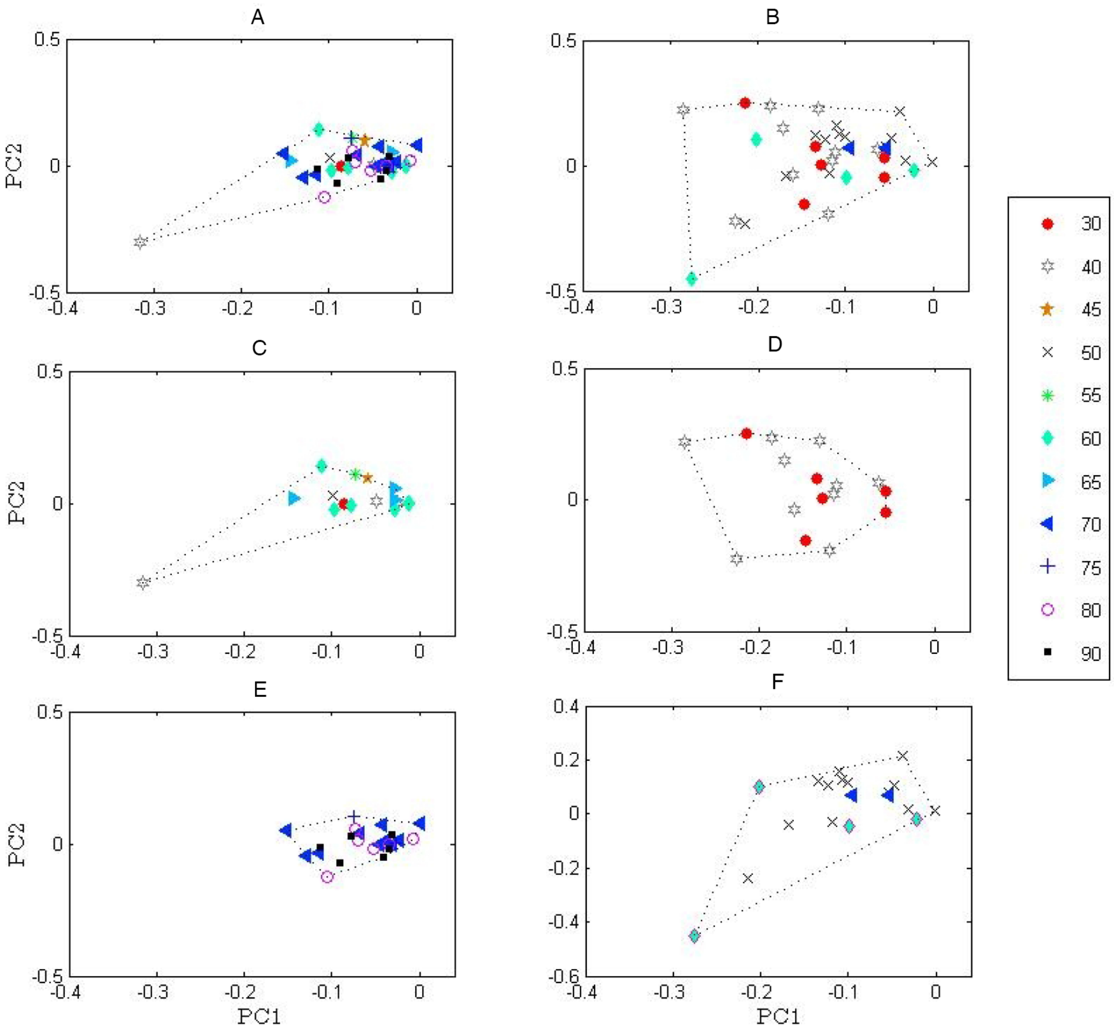

Supplementary Figure S3: Tumor cells/area difference vs. DNA copy number entropy difference by treatment arm. Top row shows the distributions of samples from CS **A.** and S **B.** in the space defined by their first two principal components obtained from the segmented data matrix. We also divided samples in each treatment arm into two groups (low and high cellularity) according to the percentage of tumor cells/area using the median as cutoff. Then, samples were again projected onto the space spanned by the first two principal components. We observed that there was no considerably difference in scatterness between the high and the low tumor cells/area groups within each treatment arm, e.g. **C.** vs **E.** and **D.** vs **F.** There was also no considerably difference in scatterness between the low tumor cells/area group in CS **C.** and the high tumor cells/area group in S **F.** Hence, we concluded that the observed difference between the treatment groups was not due to the difference in tumor cells/area but was indeed related to the treatment group specific aberration patterns present in the CGH profiles. In each panel, a point denotes a sample, and the shape and color corresponds to one of the tumor cell/area percentages shown in the legend box on the right. To complement the visualization in Supplementary Figure S3, we also conducted a formal statistical test. Namely, we tested the statistical significance of the group differences in terms of DNA copy number entropy in relation to the tumor cells/area by a simple regression. The DNA copy number was used as response variable, whereas the treatment-arm indicator and the percentage of tumor cells/area were used as independent covariates. Regression result showed that the treatment-arm indicator was a strong predictor (coeff. =0.216, p-value=0.004), while no significant association was observed for the tumor cells/areas (coeff. =-0.002, p-value=0.29). Thus, we confirmed that the observed differences between the two treatment group indeed were not due to the tumor cells/area.

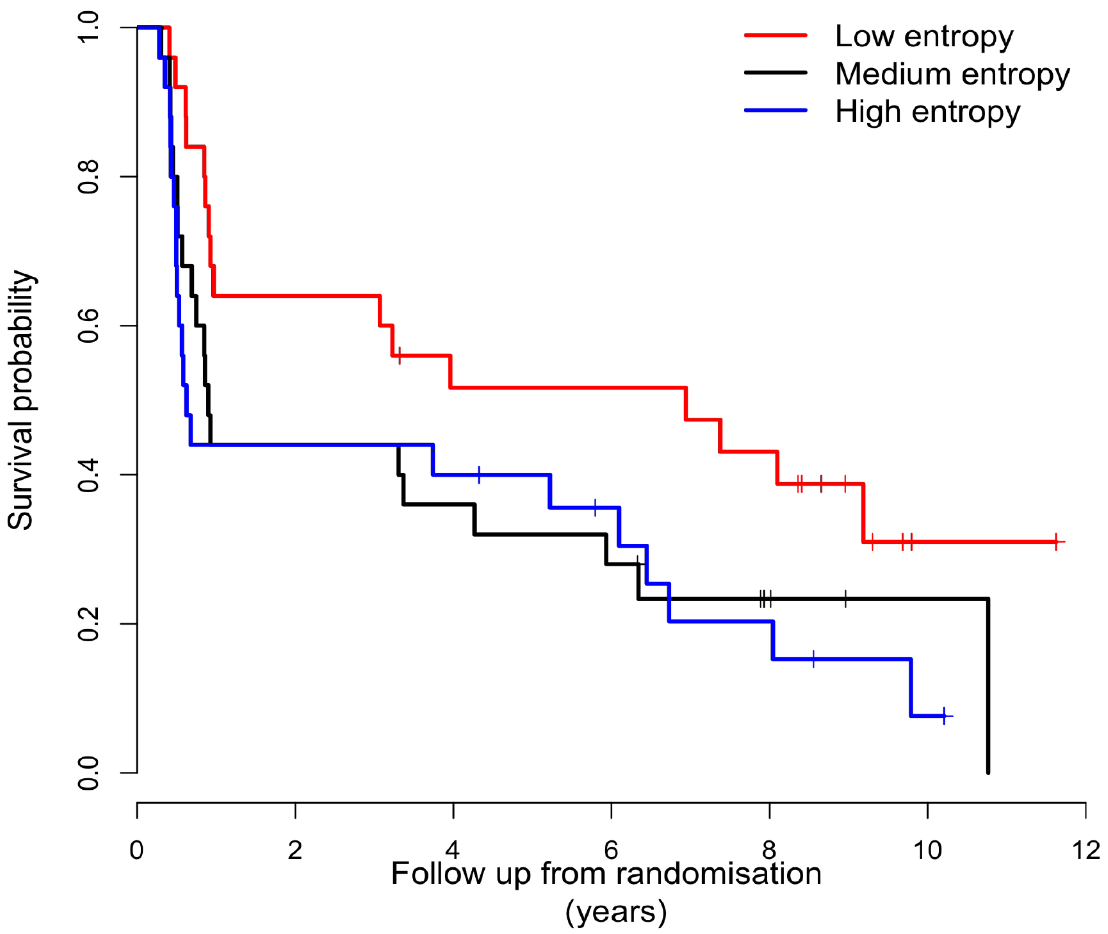

Supplementary Figure S4: Association of DNA copy number entropy with cancer specific survival. The whole cohort was divided into three equal sized patient groups based upon the DNA copy number entropy values. Patients in the low entropy group (n=25) had a better prognosis (median (range) survival time: 3.96 (0.41-11.62) years). The survival probability of patients with medium entropy (n=25, 0.90 (0.30-10.77) years) and high entropy (n=25, 0.62 (0.28-10.21) years) was similar and poorer compared to patients with low entropy.

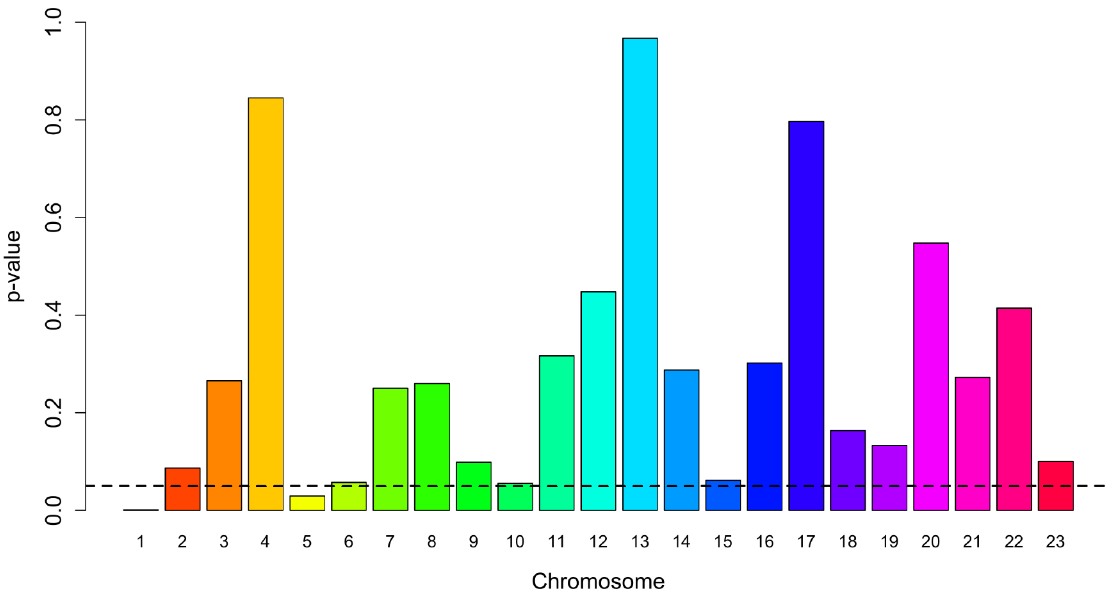

Supplementary Figure S5: Comparison of DNA copy number entropy values between treatment group stratified by chromosome. Each bar represents a chromosome. Y-axis: statistical significance quantified as p-value, X-axis: chromosomes in numerical order. The black dashed horizontal line denotes the significance threshold, p = 0.05.

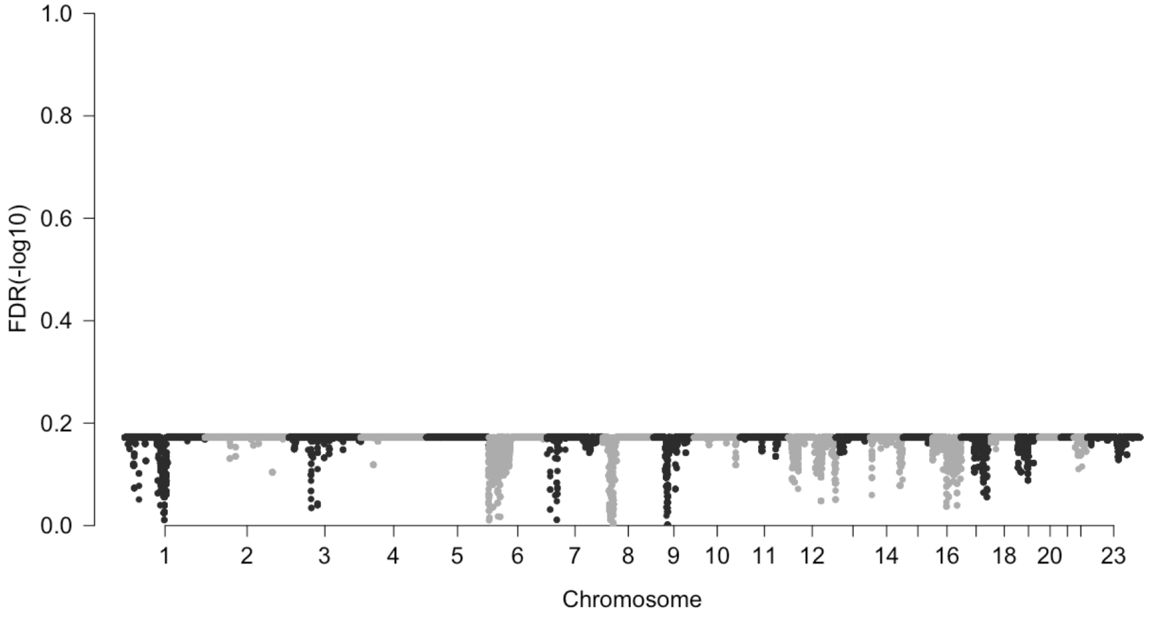

Supplementary Figure S6: Treatment group differences observed in our data. Each point in the plot denotes a feature. Y-axis: statistical significance quantified as false discovery rate (FDR), X-axis: chromosomes in numerical order. None of the feature passed the significant threshold FDR = 0.1 (-log10(0.1) = 1).

Supplementary Table S1: Association of clinopathological variables not mentioned in the paper with DNA copy number entropy.

clinicopathological variables	Association with entropy (p-value)		
	**CS+S**	**CS**	**S**
Grade of differentiation	0.655	0.056	0.551
Lymph node status (pN/ypN)	0.096	0.993	**0.038**
Depth of tumor invasion (pT/ypT)	0.096	0.074	0.910
Lymphatic channel invasion	0.059	0.426	0.112
Vnous or perineural invasion	0.596	0.067	0.475

Note that the p-value of the relationship between lymph node status and treatment group is below 0.05 for the S group. However, this is raw p-value prior to Bonferroni correction after which none of the p-values is below the signficance level.
